# Intracellular insulin in human tumors: examples and implications

**DOI:** 10.1186/1758-5996-3-5

**Published:** 2011-04-01

**Authors:** Razvan T Radulescu

**Affiliations:** 1 Molecular Concepts Research (MCR), Münster, Germany

## Abstract

Insulin is one of the major metabolic hormones regulating glucose homeostasis in the organism and a key growth factor for normal and neoplastic cells. Work conducted primarily over the past 3 decades has unravelled the presence of insulin in human breast cancer tissues and, more recently, in human non-small cell lung carcinomas (NSCLC). These findings have suggested that intracellular insulin is involved in the development of these highly prevalent human tumors. A potential mechanism for such involvement is insulin's binding and inactivation of the retinoblastoma tumor suppressor protein (RB) which in turn is likely controlled by insulin-degrading enzyme (IDE). This model and its supporting data are collectively covered in this survey in order to provide further insight into insulin-driven oncogenesis and its reversal through future anticancer therapeutics.

## Introduction

It has meanwhile been nearly a century since insulin's stimulatory effects on cell proliferation and tissue growth have been studied [1-3]. Thereby, insulin's growth-promoting actions have mainly been attributed to its complex formation with the insulin receptor located on the surface of various cells and cloned in the mid-1980s [4] along with the second messenger cascades initiated by such heterodimerization [5].

However, historically preceding and coinciding with this model on an extracellular insulin activity, there have also been reports of direct - i.e. (non-insulin receptor-mediated and) *intracellular*- insulin effects at the level of the cell nucleus [6] and, moreover, on RNA and protein synthesis by intracellular insulin [7] as well as on the transcription of immediate-early genes by intranuclear insulin [8].

In the early 1990s, this conceptual framework on an intracellular localization and action of insulin was expanded by a novel proposal according to which insulin may physically interact with the (mainly nuclear) retinoblastoma tumor suppressor protein (RB) and thereby, similar to RB-binding viral oncoproteins, inactivate RB and thus promote cell proliferation [9] which was subsequently validated experimentally [10-13], primarily in human tumor cell culture models [11-13]. The present review will focus on delineating this potential intracellular signal transduction pathway for insulin, thereby taking primarily into account human cell line and tissue studies as well as its possible inhibition by anticancer drug candidates directly targeting this molecular avenue.

## Dual mode of insulin signalling

As a result of an insulin-insulin receptor interaction in the presence of low nanomolar insulin concentrations, a second messenger cascade is activated among which the intracellular enzyme phosphatidylinositol 3-kinase (PI 3-kinase) is a major intermediary molecule [14]. Further downstream from PI 3-kinase, this cascade leads to Ras activation and, ultimately, to retinoblastoma protein inactivation through the latter's hyperphosphorylation [15], the outcome of this cascade being cell cycle progression and increased cell proliferation.

In addition to this signalling cascade initiated by insulin at the level of the cell membrane, it has become increasingly apparent over the past three and a half decades that insulin could also act as its own messenger (i.e. without the mediation of other molecules) in order to directly promote cell growth, specifically insulin molecules that are located intracellularly.

The main support for such a possibility comes from studies conducted on human cancer tissue specimens and revealing the presence of intratumoral insulin [16-19]. Intriguingly, one of these studies reported not only the detection of cytoplasmic insulin, but also of nuclear insulin [17]. These investigations indicated the possibility that such intracellular insulin may contribute to the pathogenesis of these neoplasias.

A potential mechanism for such intracellular insulin-driven tumor growth is the insulin-RB complex formation that, so far, has been experimentally demonstrated in several human carcinoma-derived cell lines [11-13]. This intracellular complex would be expected to occur primarily in the nuclei of such tumors, but a cytosolic presence of this heterodimer is also conceivable both of which subcellular localizations ought to be addressed in future studies, e.g. by employing lysates of primary tumors obtained from cancer patients.

Furthermore, the probability for this interaction should be higher in neoplastic cells which equally display a dysfunction of insulin-degrading enzyme (IDE) or, respectively, insulysin in the light of previous data showing that an inactivation of IDE leads to an increase in the nuclear localization of insulin [20]. In this context, it is interesting to note that the same compound (1,10-phenanthroline) used to block IDE activity [20] has also been shown to decrease the formation of the tumor-suppressive wild-type conformation of the p53 protein [21].

Therefore, the resulting concept on IDE as a potential tumor suppressor protecting RB from inactivation by insulin, as elaborated through structural [22-24] and proteomic [25,26] studies, should remain an additional important element to be further investigated in order to better understand cancer promotion by insulin in the years ahead. In this context, the following model may be useful in guiding such upcoming efforts (Table 1).

**Table 1:** The „IDE switch": a) surges (particularly those of a pathological nature) in the extracellular/blood level of insulin and b) defects in the activity of intracellular IDE are functionally equivalent to one another in that they both lead to an increase in intracellular insulin, the former through augmented insulin internalization [31] and the latter through decreased insulin degradation.

Extracellular/blood insulin	Intracellular IDE activity	Intracellular insulin	Intracellular insulin-RB heterodimers	Cell proliferation
Normal	Normal	Minimal	Minimal	Normal

Increased*	Normal	Increased	Increased	Increased

Normal/Increased*	Defective	Increased	Increased	Increased

*i.e. hyperinsulinemia

As a result, elevated intracellular insulin stimulates cell proliferation by binding and thereby inactivating the RB tumor suppressor, both in neoplastic diseases and in aging-related morbidities such as Syndrome X or, respectively, the metabolic syndrome which includes various clinical manifestations such as hyperinsulinemia, insulin resistance, obesity, type 2 diabetes and hypertension [32]. Interestingly, this model is supported by experimental data revealing increased intracellular insulin concentrations in monocytes from obese patients and obese diabetic patients vs. those from normal subjects [33].

In case the envisaged insulin-RB complexes and IDE dysfunction will be validated in human cancer specimens, this would then suggest as an antineoplastic treatment strategy the interference with such intracellular carcinogenesis by means of cell-penetrating peptides that bind and thereby neutralize insulin such as those peptides derived from RB and termed MCR peptides [11,13,27-30].

## Competing interests

The author declares having no competing interests.
